# Ultrastable and Responsive Foams Based on 10-Hydroxystearic Acid Soap for Spore Decontamination

**DOI:** 10.3390/molecules28114295

**Published:** 2023-05-24

**Authors:** Carolina Dari, Fabrice Cousin, Clemence Le Coeur, Thomas Dubois, Thierry Benezech, Arnaud Saint-Jalmes, Anne-Laure Fameau

**Affiliations:** 1 CNRS, INRAE, Centrale Lille, UMET, University of Lille, UMR 8207, F-59000 Lille, France; carolina.dari@inrae.fr (C.D.); thomas.dubois@inrae.fr (T.D.); thierry.benezech@inrae.fr (T.B.); 2 Laboratoire Léon Brillouin, Université Paris-Saclay, CEA-CNRS UMR CEA Saclay, F-91191 Gif sur Yvette, France; fabrice.cousin@cea.fr (F.C.); clemence.le-coeur@cnrs.fr (C.L.C.); 3 CNRS, ICMPE, UMR 7182, University Paris Est Creteil, 2 rue Henri Dunant, F-94320 Thiais, France; 4 CNRS, IPR (Institut de Physique de Rennes)-UMR 6251, University of Rennes, F-35000 Rennes, France

**Keywords:** hydroxy fatty acid, self-assembly, foam, spore, cleaning, imbibition

## Abstract

Currently, there is renewed interest in using fatty acid soaps as surfactants. Hydroxylated fatty acids are specific fatty acids with a hydroxyl group in the alkyl chain, giving rise to chirality and specific surfactant properties. The most famous hydroxylated fatty acid is 12-hydroxystearic acid (12-HSA), which is widely used in industry and comes from castor oil. A very similar and new hydroxylated fatty acid, 10-hydroxystearic acid (10-HSA), can be easily obtained from oleic acid by using microorganisms. Here, we studied for the first time the self-assembly and foaming properties of R-10-HSA soap in an aqueous solution. A multiscale approach was used by combining microscopy techniques, small-angle neutron scattering, wide-angle X-ray scattering, rheology experiments, and surface tension measurements as a function of temperature. The behavior of R-10-HSA was systematically compared with that of 12-HSA soap. Although multilamellar micron-sized tubes were observed for both R-10-HSA and 12-HSA, the structure of the self-assemblies at the nanoscale was different, which is probably due to the fact that the 12-HSA solutions were racemic mixtures, while the 10-HSA solutions were obtained from a pure R enantiomer. We also demonstrated that stable foams based on R-10-HSA soap can be used for cleaning applications, by studying spore removal on model surfaces in static conditions via foam imbibition.

## 1. Introduction

Surfactants are extensively used in a wide range of industrial applications due to their ability to adsorb at interfaces and reduce interfacial tensions. For example, they are used in food, agricultural, paint, cosmetics, and pharmaceutical applications. Surfactants can be of synthetic or biological origin; however, most of the ones used in such industries are of synthetic origin, and can potentially cause environmental and toxicological problems [1]. Therefore, the current trend is to replace synthetic surfactants with more environmentally friendly surfactants of different types [1]. This search for so-called green surfactants gives rise to a renewed interest in using fatty acid soaps in industrial applications instead of petrochemically derived surfactants [2]. Fatty acid soaps can be considered one of the oldest surfactants and are perhaps the most natural, since they are recorded as early as 2800 BCE [3]. The rise of the soap industry at the beginning of the 19th century was due to the work of Le Blanc and Chevreul, which led to the well-known saponification process of oil or fat using appropriate caustic substances [4]. In the last century, due to the modern petrochemical industry and progress in chemical processes, specific “synthetic” surfactants were developed to replace soaps in many applications. Currently, the renewed interest in fatty acid soaps comes from their many advantages: low price, present excellent biodegradability under both anaerobic and aerobic conditions, and low toxicity [2]. The most important advantage is that they are produced from renewable oils. Indeed, fatty acids are obtained by the hydrolysis of oils from various oleochemical sources. Moreover, large amount of fatty acids can be extracted and transformed into value-added new materials from the by-products of agriculture and the food industry [5]. Fatty acids have an aliphatic hydrophobic tail, which is either saturated or unsaturated, and can have some additional groups, such as the hydroxyl groups [6]. One of the most famous hydroxylated fatty acids is 12-hydroxystearic acid (12-HSA) [7]. The presence of an –OH group (chiral center) confers unique properties on the 12-HSA, which can self-assemble into fibers and tubular structures, leading to both organogels and hydrogels, and it is widely used in industrial applications for its lubricating and thickening properties [7]. The fatty acid 12-HSA is produced only from ricinoleic acid, which comes from castor oil [7].

Recently, a hydroxylated fatty acid with a very similar structure emerged in the literature: 10-hydroxy stearic acid (10-HSA), which differs from 12-HSA only in the position of the –OH group on the alkyl chain. The fatty acid 10-HSA is produced from oleic acid using microorganisms that produce an enzyme (oleate hydratase) with high enantioselectivity, leading to R-10-HSA [8,9,10,11,12,13]. Oleic acid is a very cheap starting material, compared to ricinoleic acid, as it is the most common and abundant mono-unsaturated fatty acid present in vegetable oils. Thus, it is much easier, cheaper, and better from the environmental point of view to produce 10-HSA, compared to 12-HSA. Many studies in the literature describe and compare the different ways to produce 10-HSA from microorganisms but, to the best of our knowledge, there is no previous study on the surfactant properties of 10-HSA soap. Only nominal data are available on the morphology of the self-assembled structures and gelling properties of non-aqueous solvents [14].

Our aim in this research was to study the self-assembled properties of a model 10-HSA soap system in bulk aqueous solution and the resulting foaming properties, in order to compare the results with 12-HSA soap, for which there exists a large corpus of knowledge in the literature. Therefore, to disperse the 10-HSA in water and to compare it with 12-HSA, we used the same strategy based on the use of monoethanolamine (MEA), a model organic counter-ion, to produce fatty acid soap with a stoichiometric molar ratio between 10-HSA and MEA [15,16,17]. We used a multiscale approach to determine the self-assembled and surface properties of 10-HSA by combining microscopy techniques, small-angle neutron scattering (SANS), wide-angle X-ray scattering (SAXS), rheology experiments, and surface tension measurements as a function of temperature. We compared the respective behaviors of 10-HSA and 12-HSA at different length scales. We also demonstrated how foams produced by 10-HSA soap can be used for cleaning and decontamination applications, taking advantage of their stability to remove model contaminants (spores) from model surfaces. 

## 2. Results and Discussion

### 2.1. Critical Aggregation Concentration of 10-HSA Dispersion

**Figure 1:**
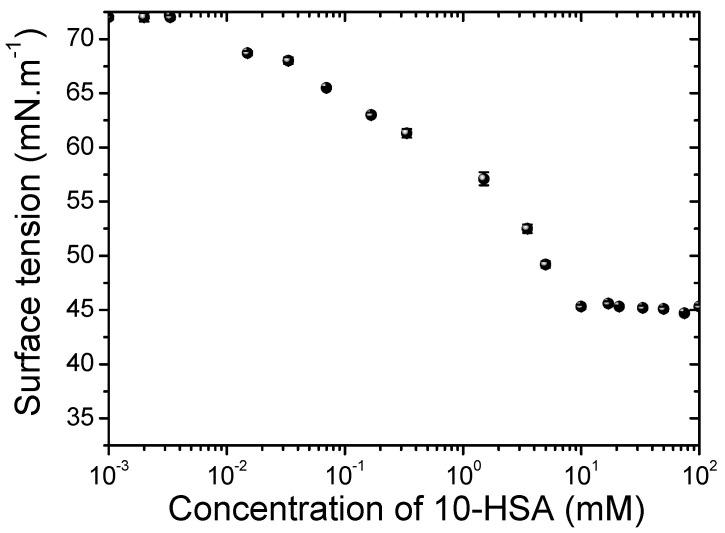
The surface tension of aqueous solutions of 10-HSA/MEA as a function of concentration at T = 20 ± 1 °C.

First, we studied the critical aggregation concentration (CAC) of 10-HSA dispersion. The surface tensions of the aqueous 10-HSA/MEA solutions were determined as a function of 10-HSA concentration at T = 20 ± 1 °C. The CAC was determined to be around 10 mM (3.3 mg·mL^−1^), associated with a plateau of surface tension at 45 N·m^−1^ ± 0.2 (Figure 1). The CAC was higher for 10-HSA than the values reported for 12-HSA [15,18]. Moreover, the surface tension reached above the CAC was much lower for 12-HSA than for 10-HSA. For example, at 10 mg·mL^−1^, the surface tension value was around 23 mN·m^−1^ for 12-HSA [19]. We hypothesize that the difference in CAC and surface tension values between 12-HSA and 10-HSA may originate from either the position of the –OH group on the alkyl chain or/and the chirality since the 12-HSA was studied in a racemic mixture. The chirality of 12-HSA on the molecular scale is known to have a strong effect at much larger scales and controls the self-assembled structural and interfacial properties [7]. Indeed, it was shown in the literature that the presence of a racemic mixture for 12-HSA has a stronger effect on the surface properties than the difference of two carbons for the –OH position between 12-HSA and 10-HSA [20,21,22]. 

### 2.2. Self-Assembled Structure and Rheological Properties at 25 °C

**Figure 2:**
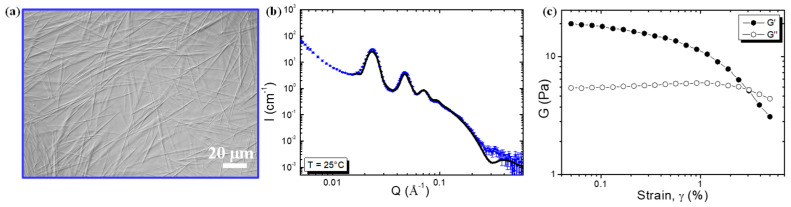
(**a**) Phase contrast microscopy picture of 10-HSA at 25 °C. (**b**) SANS intensity profile of 10-HSA at 25 °C. The black line corresponds to the best fit of the data described in the Supplementary Materials. (**c**) Oscillatory measurements of 10-HSA, elastic *G*′ (●), and viscous *G*″ (○) moduli plotted as a function of the strain amplitude, where *γ* is a constant *f* = 1 Hz at 25 °C.

The concentration of 10-HSA was fixed at 10 mg·mL^−1^ so as to be above the CAC (at around 3 times the CAC) and to compare the results with previous studies performed with 12-HSA at this concentration [16,19,23]. At room temperature, a homogenous turbid phase with birefringence was observed. By using phase contrast microscopy, the presence of micron-sized rods was observed (Figure 2a). These rods were very similar in shape and in length and size to those obtained using 12-HSA [17]. In order to gain insight into the local structure of these rods at the nanometric scale, SANS experiments were performed. This technique does not provide information on the total size of the tubes observed via microscopy, but it does allow us to elucidate the self-association of fatty acids on a scale of up to a hundred nanometers. Figure 2b displays the scattering profile. In the low *Q* region, four sharp peaks were observed. Their positions were in a ratio of 1:2:3:4 (*Q*_0_, 2*Q*_0_, 3*Q*_0_, 4*Q*_0_). The presence of the first strong correlation peak, followed by its harmonics, showed that the inner rods were formed from periodically stacked bilayers, separated by water layers. Above *Q* = 0.02 Å^−1^, the slope in *Q*^−3^ showed the presence of the large objects observed previously: the rods. The 10-HSA bilayers arrange themselves into multilamellar tubes, as do the 12-HSA bilayers. The interlamellar spacing, corresponding to one lipid bilayer plus one water layer in the stack of bilayers, was determined by using the position of the first correlation peak at 0.0237 Å^−1^. It was equal to 2π/*Q*_0_ = 263 Å, which was lower than for the 12-HSA self-assemblies, for which a value of around 320 Å was obtained in the same experimental conditions of ethanolamine/fatty acid ratios and concentrations [16]. The swelling of the bilayer was lower for 10-HSA than for 12-HSA. From the SANS fitting (see Figure S1 in the Supplementary Materials for details), we determined both the number of stacked bilayers and the Caillé parameter, while accounting for the thermal fluctuations of the bilayers [24]. From the fitting, we found that the Caillé parameter was around 0.06, with 5 bilayers. In the case of 12-HSA, the Caillé parameter was around 0.1, with 4 bilayers [15]. For the 10-HSA, the bilayers were more rigid than those of the 12-HSA, most probably due to the fact that the molecules are more organized when they are not in a racemic mixture. 

In the high *Q* region, the bilayer thickness was determined from the oscillation of the form factor (Figure S1a) at around 20.5 Å. This thickness is less than twice that of the 10-HSA molecule in its extended conformation (21 Å), suggesting that the bilayers could be either in the L*β* gel state, with its interdigitated alkyl chain, or in the L*α* fluid state [17]. To determine the 10-HSA alkyl chain state of crystallinity, we then performed a wide-angle X-ray scattering experiment (WAXS). Four diffraction peaks were observed in the WAXS diffractogram (Figure S1b), located respectively at 1.37, 1.47, 1.55, and 1.59 Å^−1^. We deduced that the 10-HSA was mostly in a triclinic conformation and that the alkyl chains were in a gel state. This result was different from the 12-HSA under the same experimental conditions since the bilayer was in a gel state but had a bilayer thickness of around 42 Å, which was twice the length of the 12-HSA molecule and, therefore, not interdigitated, and with an orthorhombic conformation [6]. This difference between the two systems may, again, originate from its chirality, since the 12-HSA under study was in a racemic mixture and the 10-HSA was not (R-10-HSA). Chirality has already been shown to have a strong effect on alkyl chain packing in the alkane system [25]. 

Oscillatory tests were performed to determine the rheological behaviors of the 10-HSA aqueous dispersion of multilamellar tubes at 25 °C. The results of the amplitude sweeps (at a fixed frequency *f* = 1 Hz) are shown in Figure 2c. The dispersion was viscoelastic, with viscous and elastic moduli (*G*′ and *G*″) depending on the applied amplitude. At low amplitude, *γ* (below 3%), *G*′ was higher than *G*″, but at the highest amplitude, *G*″ was higher than *G*′. At a low *γ*, there was gel-like behavior of the multilamellar tubes. As the amplitude was increased, a yield point appeared, and the dispersion started to flow above a typical yield strain of *γ* = 1% (the value at which *G*′ started to rapidly decrease). This behavior is fully reminiscent of that of the 12-HSA dispersions, where only the values of *G*′ and *G*″ were slightly higher, and the yield strain was at 0.5% [19]. We suggest that this difference could come from the length or/and or the diameter of the tubes [19].

### 2.3. Evolution of the Self-Assembled Structure and Rheological Properties with Increasing Temperature

**Figure 3:**
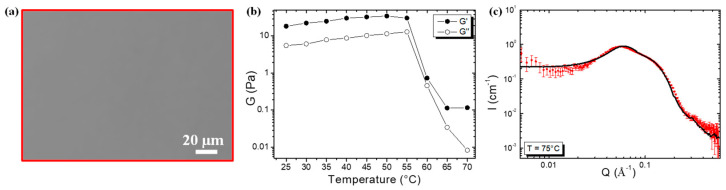
(**a**) Phase contrast microscopy picture of 10-HSA at 65 °C. (**b**) Variations of elastic G′ (●) and viscous G″ (○) moduli upon heating. The moduli were measured at γ = 0.1% and *f* = 1 Hz. (**c**) SANS intensity profile of 10-HSA at 75 °C. The line corresponds to the best fit of the spectrum described in the Supplementary Materials.

By increasing the temperature, we observed that around 65 °C, the turbid sample turned into a limpid sample. Using phase contrast microscopy, no rods were visible any longer (Figure 3a). In order to determine precisely the temperature transition threshold, we studied the evolution of the elastic and viscous moduli, *G*′ and *G*″, respectively, monitored as a function of the amplitude of deformation and at a fixed frequency for various temperatures (see Figure S2 in the Supplementary Materials). In Figure 3b, the quantitative dependence on temperature for *G*′ and *G*″ found in the low amplitude plateau below the yielding point (a low amplitude of 0.1% and a frequency of 1 Hz) is represented. Below 60 °C, when multilamellar tubes were present in the solution, we observed that both the viscous and elastic moduli remained almost constant at around 20–40 Pa for *G*′ and around 6–10 Pa for *G*″. At 60 °C, a sharp decrease in both *G*′ and *G*″ was observed, down to very low values at 65 °C, which was even below the limit of resolution for *G*′. This strong variation in the moduli over only a few degrees centigrade confirms that the temperature transition was at around 65 °C from a rheological point of view. Due to the experimental limit of the rheometer, we did not observe the crossover between *G*′ and *G*″ during the transition. The SANS spectrum was recorded above this transition, at 75 °C (Figure 3c). It no longer shows the typical features of a lamellar phase but instead displays the characteristic features of spheres, interacting through repulsive interactions. At a high *Q* value, the spectrum was fitted with a form factor of a sphere and a structure factor with a radius of 21 Å that corresponds to the length of the fatty chain (Figure S1c in the Supplementary Materials). Therefore, spherical micelles were present inside the limpid solution. At a low *Q* value, in the SANS spectrum, a decrease in scatter intensity was observed when moving toward the low *Q*. This decrease comes from the low isothermal compressibility of the system since the 10-HSA micelles were mainly composed of the ionized 10-HSA molecules, which were negatively charged. Therefore, the spherical micelles repelled each other due to electrostatic repulsion over large distances, which gave rise to the appearance of a broad correlation peak. In the direct space, the position of the broad correlation peak corresponded to the average distance between the spherical micelles. The position of the broad correlation peak at *Q* = 0.058 Å corresponded to a mean distance between micelles of around 108 Å. The 10-HSA multilamellar tubes exhibited a transition into spherical micelles above 65 °C.

By decreasing the temperature from 70 °C to 25 °C, the rods appeared to be back when examined with optical phase contrast microscopy, showing that the self-assembled transition was reversible. The 10-HSA multilamellar tubes transited into spherical micelles in the same way as for 12-HSA multilamellar tubes, as depicted in an earlier study [17], but this was at a lower temperature (65 °C for 10-HSA and 70 °C for 12-HSA). The sizes of the spherical micelles were similar, as well as the mean average distance, given that the fatty acid concentration was the same. At high temperatures, the size of the self-assembled structures in the solution was mainly linked to the size of the molecules, which was similar in both 12-HSA and 10-HSA, and the presence of the racemic mixture no longer played a role.

### 2.4. Foaming Properties with Time and Temperature

**Figure 4:**
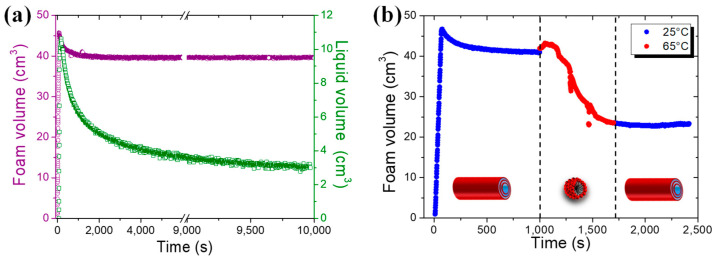
(**a**) Evolution of the foam volume (purple) and of the liquid volume in the foam (green) over time at 25 °C. (**b**) Evolution of the foam stability by measuring the evolution of foam volume over time at different temperatures: at 25 °C when multilamellar tubes were present and at 65 °C when spherical micelles were present.

The foaming properties of 10-HSA dispersion were evaluated for two different ways of producing foams: shaking by hand and bubbling gas through the tube dispersion. A large volume of foam can be produced from the 10-HSA multilamellar tube dispersion via shaking by hand, demonstrating the high foamability of the dispersion (Figure S3 in the Supplementary Materials). This foam was shown to be very stable with time when kept at room temperature for one month (Figure S3). To quantify the foaming properties in terms of foamability, stability with temperature, and evolution of the liquid fraction, we produced foams by bubbling gas into the 10-HSA aqueous dispersion at 25 °C (Figure 4). The fixed foam volume to reach was 45 cm^3^ with a gas flow rate fixed at 35 mL·min^−1^. It was reached in around 75 s, showing that all the gas was incorporated inside the foam. Therefore, the foamability was optimal and the gas bubbles were quickly and efficiently stabilized (Figure 4a). Then, the foam volume remained constant with time throughout the experiment, confirming the ultrastable character of the foam. In terms of the liquid fraction, at the end of bubbling, the average liquid fraction was around 24%, corresponding to a so-called wet foam. The liquid drained quickly out of the foam in the first few minutes, then the liquid fraction stabilized at around 7.5%. This foaming behavior was very similar to that obtained for 12-HSA multilamellar tube dispersions [23]. Microscopically speaking, we surmise that the foams are first stabilized by the 10-HSA monomers released from the tubes at the gas–solution interface and that these monomers protect the bubbles and provide some stability to the films separating the bubbles. In parallel, the crowding of the multilamellar tubes within the liquid foam channels stabilizes this liquid skeleton and prevents further destabilization. At this stage, the foams based on 10-HSA and 12-HSA appeared very similar in terms of their foaming properties since the foaming properties are mainly linked to the presence of tubes at the micron scale and to the number of tubes, which is similar. 

Then, we studied the foam’s stability behavior as a function of temperature, below and above the temperature threshold transition between tubes and spherical micelles (Figure 4b and Figure S4 in the Supplementary Materials). At 25 °C, when multilamellar tubes were present within the foams, the foam volume remained stable over time (Figure 4b). However, when the foam temperature was increased to 65 °C, the transition of tubes into micelles inside the foam liquid channels led to a rapid foam volume decrease from 40 cm^3^ to 25 cm^3^. By switching back the temperature quickly to 25 °C, the decrease in the foam volume was stopped; the foam volume remained constant over time because the micelles transited back to tubes inside the foam (Figure 4b). The 10-HSA dispersion led to thermoresponsive foams in the same way as that previously described elsewhere for 12-HSA dispersion [23]. Only the temperature at which the foam stability could be switched between stable and unstable states was different since it was driven by the temperature transition threshold between multilamellar tubes and spherical micelles.

### 2.5. Cleaning of Spore Contamination on Surfaces by Foam Imbibition

**Figure 5:**
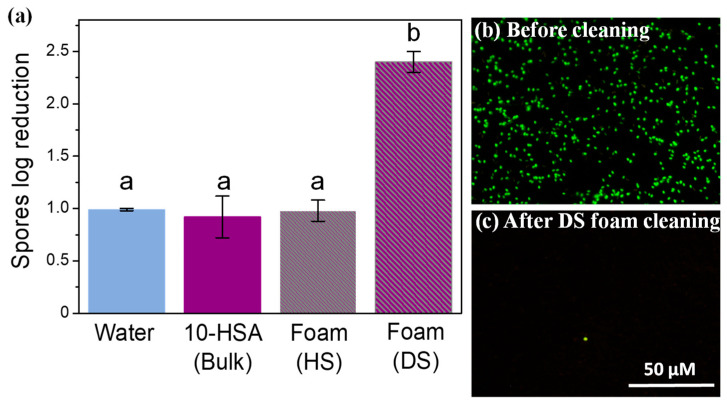
(**a**) Spore log reduction on the model surface after 30 min for water, 10-HSA dispersion, hand-shaken (HS) foam, and double-syringe (DS) foam. The small letters, a and b, indicate groups of statistical differences according to Tukey’s test (*p* < 0.05). (**b**) Epifluorescence microscopy pictures of the model surface contaminated by fluorescent spores before cleaning, and (**c**) after 30 min of cleaning with DS foam. The scale bar represents 50 µm in all the pictures.

Then, we took advantage of the 10-HSA foam’s stability to study the potential of these foams for decontamination applications [26]. We used stainless-steel plates as model surfaces and spores from *Bacillus subtilis* (size 400–700 nm) as the model contaminant to study the absorption process as a result of imbibition inside the foam [27]. The model surfaces were kept in a horizontal position and the foams were put in direct contact with the contaminated surface (see Section 3.2). To favor foam imbibition and avoid the detachment of the spores from the surface induced by drainage and ensure that the plates remained underneath the foam together with plenty of liquid, we decided to first let the foam dry for 10 min, and then put the foam on the surfaces contaminated with spores. We produced foams using the two different foaming techniques to vary the bubble size and the liquid fraction, which are the main parameters governing imbibition. Thus, we produced foams using the hand-shaking (HS) method and the double-syringe (DS) method [28]. Before cleaning, the average bubble radius was 103 ± 69 µm and 19 ± 8 µm for the HS foam and DS foam, respectively. The remaining average liquid fraction of the foams after 10 min was around 10 ± 0.4% and 19 ± 0.8% for the HS foam and DS foam, respectively. It is important to note that the liquid fraction was most probably not uniform throughout the foam, with drier foams at the top than at the bottom. The foams remained in contact with the contaminated surfaces in a horizontal position for 30 min. First, we checked the bubble size evolution after 30 min of cleaning. The bubble size slightly increased, to reach 129 ± 88 µm and 19 ± 8 µm for the HS foam and DS foam, respectively. Multilamellar tubes were clearly observed inside the foam before and after cleaning (Figure S5 in the Supplementary Materials). Then, the stainless-steel plates were removed, and the effect of contact with the foam was quantified by measuring the spore log reduction after 30 min, as shown in Figure 5a. The spores’ log reduction was around 1.13 ± 0.14 for the water due to the hydrophilicity of the spores, as already observed previously, which could be removed by water [29]. The spores’ log reduction for the pure 10-HSA dispersion was 0.92 ± 0.20, which was similar to water. It is important to point out that the 10-HSA dispersion did not have an effect on the spores’ viability, which was similar between the water and 10-HSA dispersion. The spores log reduction for the 10-HSA foams was 0.98 ± 0.10, and 2.40 ± 0.10, respectively, for HS and DS. The HS foam had a similar effect to that of just water or the 10-HSA dispersion regarding spore removal. However, the spores’ log reduction value was much higher for the 10-HSA DS foam, showing that this foam could efficiently remove spores from the plate. Due to their small size compared to the foam’s liquid channels and plateau borders, the spores could be removed efficiently by the foam. The effect of foam cleaning on spore removal was also assessed by epifluorescence microscopy before and after foam cleaning (Figure 5b). Before foam cleaning, the fluorescent spores could be detected easily on the model surfaces. After cleaning with DS foam, almost no spores could be observed, confirming the results of spore log reduction quantification. 

**Figure 6:**
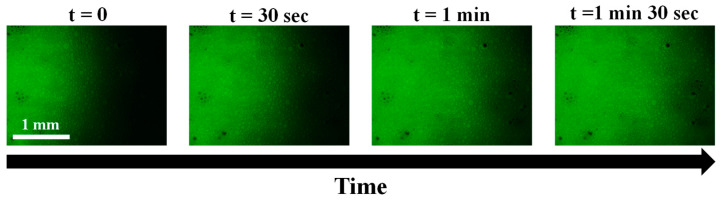
Epifluorescence microscopy pictures of the penetration of the aqueous dispersion of spores into a double-syringe foam over time. Here, t = 0 corresponds to the image taken just after contact with the foam. The scale bar represents 1 mm in all the pictures.

To visualize and confirm how the spores could be soaked up by the DS foam, using fluorescence microscopy, we assessed the spores’ penetration dynamic into the 10-HSA DS foam, as already reported in the literature regarding oil imbibition in foams (Figure 6) [30,31,32]. Immediately after the initial contact of the spores’ suspension with the foam, we observed the penetration of the fluorescent spores inside the foam without destroying the foam. Therefore, the high spore log reduction for DS foam came from the foam imbibition phenomenon, resulting in the very rapid detachment of the adhering spores and their penetration inside the foam. The difference between HS and DS foam could be explained by the bubble size difference (a factor of ten between HS and DS foam bubble sizes) since it is known that the smaller the bubble size, the smaller the liquid channel section, and the greater the capillary pressure and the imbibition phenomenon [26,33]. In our case, we assume that even if the average liquid fractions were different, they were still within the range of wet foams, and we also assume that only the bubble size had an effect on foam imbibition [32]. Moreover, by comparing the log reduction obtained for the DS foam to the log reduction obtained for foam flow in dynamic conditions using the same spore model and the same surface, we observed that the values are similar, showing that foams in static conditions are very effective for efficiently removing spore contamination, thanks to the imbibition process [29].

## 3. Materials and Methods

### 3.1. Materials 

#### 3.1.1. Preparation of 10-Hydroxystearic Acid Dispersion

Monoethanolamine was purchased from Sigma Aldrich (Saint Quentin Fallavier, France) and was used as received. The 10-HSA was obtained using the following method [34]. The 10-HSA was weighed precisely in a tube, then ultrapure water was added so that the concentration was 10 mg·mL^−1^ (1 wt % in water). Next, the desired volume of a 1 mol·L^−1^ monoethanolamine solution prepared in MilliQ water was incorporated to reach equivalence (molar ratio = *n*_monoethanolamine_/*n*_10-HSA_ = 1, with *n* representing the molar concentration in mol·L^−1^). The mixture was heated at 80 °C for 15 min until all the 10-HSA powder was fully dispersed and was then vortexed. Samples were further stored at 4 °C and, prior to use, they were heated again to around 80 °C for 5 min and then cooled to room temperature.

#### 3.1.2. Preparation of Solid Model Surfaces

All the cleaning experiments were carried out on rectangular (45 mm × 15 mm) AISI 316 stainless-steel plates (APERAM, Isbergues, France) with a 2R factory finish. In order to have similar surface properties to those found in the food industry, the surfaces were subjected to a conditioning process that has been described previously [29]. In brief, the plates were immersed in milk at room temperature for 30 min and then they were rinsed for 5 min under osmosis water by overflow. The plates were immersed in a 0.5 wt % sodium hydroxide solution at 70 °C for 30 min and then they were rinsed in osmosis water for 5 min. This cycle was repeated 15 times. Then, they were fouled with *Escherichia coli* strains that have previously been shown to produce biofilms on stainless-steel surfaces. Afterward, the plates were subjected to cleaning and disinfection steps. Twenty-four hours before each experiment, the plates were sterilized in a dry-heat oven at 180 °C for 1 h.

#### 3.1.3. Preparation of Hydrophilic Spore Suspension

*Bacillus subtilis* PY79 is a laboratory strain of bacteria that is known to produce hydrophilic spores. The *Bacillus subtilis* PY79 strain was tagged with green fluorescent proteins to make the spores fluoresce. The method of producing the spores has been described previously elsewhere [27].

### 3.2. Methods 

#### 3.2.1. Determination of Critical Aggregation Concentration via Surface Tension Measurements 

Measurement of the air-solution surface tension as a function of the concentration of 10-HSA/monoethanolamine dispersion was performed using an automatized surface tension plate reader, the Kibron Delta-8 (Kibron, Helsinki, Finland). A volume of 50 µL of aqueous dispersion was placed on the 96-hole platform. Measurements were performed at 20 °C after a waiting time of 10 min to ensure equilibrium at the air–water interface. Calibration was performed using ultrapure water at 20 °C. Measurements were performed three times for each concentration.

#### 3.2.2. Microscopic Observation of the 10-HSA Dispersion 

Phase contrast microscopy observations were performed at different temperatures (25–75 °C) at 40× magnification, using an optical microscope in the phase contrast mode (Nikon Eclipse E-400) equipped with an STC-CM202 USB color camera (Sentech, Singapore). 

#### 3.2.3. Rheological Measurements

An Anton Paar MCR301 rheometer was used to study the viscoelastic properties of the dispersion. Experiments were performed with a cone-plate cell setup. The walls of the tools that were in contact with the samples were rough, to avoid wall slips. The rheometer was equipped with a Peltier system to control the temperature.

#### 3.2.4. Small-Angle Neutron Scattering (SANS) and Wide-Angle X-ray Scattering (WAXS)

SANS experiments were performed at the Laboratoire Léon Brillouin (Saclay, France). Samples were prepared with deuterated water and were held in flat quartz cells with a 2 mm optical path length for the sample at a concentration of 10 mg·mL^−1^ (1 wt %). A PAXY diffractometer was used for the SANS experiments. Four configurations were chosen to get a Q-range that lies between 2·10^−3^ and 5·10^−1^ Å^−1^ (*λ* = 5 Å, D = 1 m; *λ* = 5 Å, *D* = 3.5 m; *λ* = 8 Å, *D* = 5 m; *λ* = 15 Å, *D* = 7 m, respectively) with a significant overlap between the three configurations. The temperature was controlled by a circulating fluid to within ± 0.2 °C. The neutron wavelength was set to the desired value with a mechanical velocity selector (*Δλ*/*λ* ≈ 0.1). The azimuthally averaged spectra were corrected for the solvent and empty cells, as well as for background noise, and were normalized to the inconsistent H_2_O signal using the PASINET software package provided at the beamline. The fitting software used was SasView 5.0.4 (http://www.sasview.org/). The fitting models are detailed in the Supplementary Materials.

The WAXS experiments were carried out with a Xeuss 2.0 instrument from Xenocs (Grenoble, France) using a micro-focused sealed-tube Cu K-alpha source with a wavelength of 1.54 Å. The temperature was fixed at 25 °C. The dispersion was held in a 1.5 mm glass capillary tube.

#### 3.2.5. Foam Characterization with Temperature and Time

The foams were produced with the FoamScan apparatus (IT CONCEPT, Longessaigne, France). Foam was generated in a round glass column (21 mm in diameter) by sparging N_2_ gas through a fixed volume (12 mL) of the 10-HSA dispersion via a porous disk (pore size 10–14 µm) located at the bottom of the glass column. The flow rate was fixed at 35 mL·min^−1^. The FoamScan uses image analysis to monitor foam formation and stability with a CCD camera (Sony Hexwave HAD). For all our measurements, a foam volume of 45 mL was generated. The temperature of the glass column was controlled using a water bath. The liquid volume in the foam was assessed by means of conductivity measurements.

#### 3.2.6. Production and Characterization of Foams for Spore Detachment

The foams were produced at room temperature using two techniques to change the liquid fraction and the bubble size: the hand-shaking and double-syringe methods. For the hand-shaking foam method, we put 15 mL of the 10-HSA dispersion in a 50 mL cylindrical graduated plastic container (Falcon tube 50 mL, internal diameter 2.5 cm, 11.5 cm in height). Then, the dispersion was agitated for 15 s to produce the foam, and it was always produced by the same operator. For the double-syringe foam method, two 12-mL syringes were connected with a Luer-lock connector. One syringe was filled with 3.6 mL of cleaning solution and 8.4 mL of air. The second syringe was maintained with the piston in the fully closed position. The foam was then produced by pushing the plungers of the connected syringes 30 times by hand. Then, the foam thus produced was placed in a 50 mL Falcon tube. In order to have enough foam to cover the plates, it was necessary for the double-syringe technique to produce foam more than once (three times), then the resulting foams were mixed together in the 50 mL tube. Once both foams were produced, they were left for 10 min for the liquid in the foams to drain, after which we stabilized the liquid fraction. Then, we removed the drained liquid by drilling a small hole in the Falcon tube with a thumbtack. The final liquid fraction was determined using Equation (1):

(1) $$\emptyset =\left(V_{initial\;liquid}-V_{drained\;liquid}\right)/\;V_{foam}$$

where *V_initial liquid_* corresponds to the initial amount of liquid; *V_drained liquid_* corresponds to the amount of liquid drained after 10 min; *V_foam_* corresponds to the volume of foam after 10 min.

The bubble size in the foams produced before and after the cleaning tests was determined by optical microscopy (Zeiss Axioskop 2 Plus, Oberkochen, Germany), following the method introduced by Gaillard et al., using ImageJ software [35]. We used the fluorescence mode of the same microscope to follow the imbibition of fluorescent spores in the foam. A volume of 100 µL of spore suspension was carefully injected between the microscopic slides containing the foam. All experiments were carried out in triplicate.

#### 3.2.7. Viability of Spores

We carried out serial dilutions of the spore suspension in sterile Milli-Q water and in the 10-HSA dispersion. The dilutions were then plated in tryptic soy agar (TSA; Biokar Diagnostics, Allonne, France) and the plates were incubated for 24 h at 30 °C. The number of colony-forming units (CFU) was counted manually and the results were expressed as CFU·mL^−1^.

#### 3.2.8. Spore Detachment Analysis

**Figure 7:**
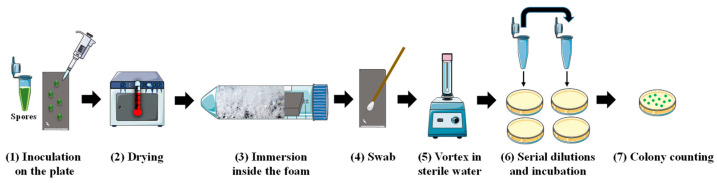
Schematic showing the methodology for spore removal on stainless-steel surfaces using foam. (1) Five drops (1 µL) of the spore suspension were placed on the surface of the model plate with a micropipette; (2) the plates were dried in an oven for 1 h at 30 °C; (3) the soiled plates were then placed in the tubes containing the foams and were kept in a horizontal position for 30 min; (4) each plate was sampled with a dry cotton swab; (5) the swab was put in a tube with 5 mL of sterile Mill-Q water and vortexed for 1 min at 2400 rpm; (6) serial dilutions were made in sterile Milli-Q water for each tube and were then placed in tryptic soy agar and incubated for 24 h at 30 °C; (7) the number of colony-forming units (CFU) was counted manually.

The spore detachment analysis protocol was adapted from the literature and is summarized in Figure 7 [29,36]. The spore suspension was diluted in sterile Milli-Q water to obtain a concentration of 10^8^ CFU·mL^−1^. In order to avoid the presence of spore aggregates, the spore suspension was sonicated for 2 min 30 s in an ultrasonic bath (Bransonic 2510E-MT, Branson Ultrasonics Corporation, Danbury, CT, USA) before use. Five drops of 1 µL of the spore suspension were placed on the surface of each plate with a micropipette (Figure 7). The plates were then dried in an oven for 1 h at 30 °C. The soiled plates were then either placed in the tubes containing the foam (one plate per tube), or in 15 mL of the cleaning solution, or in 15 mL of pure water, and they were kept in a horizontal position for 30 min. A soiled plate was used as a control for the quantification of the initial spore concentration in each experiment.

To isolate the adhered spores, each plate was sampled with a dry cotton swab (Copan, Brescia, Italy), which was then placed in a tube containing 5 mL of sterile Milli-Q water. Each tube was vortexed for 1 min at 2400 rpm and then the swabs were removed from the tubes. For the quantification of the spores, serial dilutions were made in sterile Milli-Q water for each tube and were then plated in tryptic soy agar (TSA; Biokar Diagnostics, Allonne, France). The plates were incubated for 24 h at 30 °C and the number of colony-forming units (CFU) was counted manually. The results were expressed as *CFU·plate^−1^*. The foams’ cleaning efficiency was calculated by dividing the number of viable spores after the cleaning tests by the number of spores on the plates used as a control for the initial spore concentration. The cleaning efficiency was expressed in terms of log reduction using Equation (2):

*Log reduction* = *log* (*CFU·plate*^−1^_*t*30*min*_ − *CFU·plate*^−1^_*t*0_) (2)

where *CFU*·*plate*^−1^*_t_* is the number of colony-forming units per plate after cleaning (*t* = 30 min); *CFU*·*plate*^−1^*_t_*_0_ is the initial number of the colony-forming units per plate. All the experiments were carried out in triplicate.

The spore drops placed on the plate surfaces were also observed before and after cleaning, using the same epifluorescence microscope as described previously. For each plate, 4 pictures were taken. The experiments were carried out in duplicate.

#### 3.2.9. Statistical Analysis

All the experiments were carried out in triplicate and the results were expressed as the mean ± standard deviation. The results were compared using a one-way analysis of variance and Tukey’s test to analyze the statistical differences (*p* < 0.05). The analysis was performed using SAS V8.0 software (SAS Institute, Gary, NC, USA).

## 4. Conclusions

For the first time in the literature, we provide results concerning the behavior of 10-HSA as a soap in aqueous dispersion. Our study showed that 10-HSA, in the presence of monoethanolamine with a molar stoichiometric ratio, led to the formation of micron-size multilamellar tubes in the aqueous dispersion, which transited into spherical micelles at a high temperature above a temperature threshold. This behavior is similar to that of 12-HSA molecules solubilized in water in the same experimental conditions, with the same protocol [15,16,23]. However, the structure at the nanoscale, the thermal behavior, and the interfacial properties are different between 10-HSA and 12-HSA [19,37]. Such a difference likely comes from the use of a racemic mixture for 12-HSA in the previous studies, whereas the 10-HSA molecules under scrutiny herein were just based on the R-enantiomer [7,15]. Our results thus confirm the utmost importance of chirality on the self-assembled structures and their thermal behavior, as already observed in the case of 12-HSA organogels [7]. The position of the –OH group on the alkyl chain also plays a role. In the future, it would be interesting to compare R-12-HSA with the results obtained here using 10-HSA. In addition, given that the 10-HSA produced micron-size multilamellar tubes, as with 12-HSA, their aqueous dispersions can be used to design ultra-stable foams with a threshold temperature of destabilization triggered by the tube/micelle transition [23,38,39]. These stable foams are good candidates for cleaning or decontamination applications, as shown here for spore removal. The efficiency of the decontamination could be improved by further decreasing the bubble size and the liquid fraction, or by adding a biocide agent [40]. We hope that our study will open the door for various applications wherein 10-HSA fatty acids could replace 12-HSA fatty acids. Indeed, unlike 12-HSA, which is difficult to obtain as it comes only from the ricinoleic acid in castor oil, 10-HSA can be easily obtained, either from oleic acid and commercially available baker’s yeast, or from the action of fatty-acid hydratase, which is found in many common probiotic microorganisms [7,11,13]. 

## Data Availability

The raw data will be available from the corresponding author upon reasonable request.

## Supplementary Materials

The following supporting information can be downloaded at: https://www.mdpi.com/article/10.3390/molecules28114295/s1. Figure S1: SANS data and WAXS data. Figure S2: Rheological data as a function of the temperature. Figure S3: Foam stability over time. Figure S4: Foam stability according to temperature. Figure S5: Microscopic images of foam used for cleaning.
