# Supplementary material for: Ultrastable and Responsive Foams Based on 10-Hydroxystearic Acid Soap for Spore Decontamination
Source: Molecules. 2023 May 24;28(11):4295. doi: 10.3390/molecules28114295 (PMC10254499; doi:10.3390/molecules28114295)
Supplement: Supplementary file 1 [file molecules-28-04295-s001.zip › molecules-2396167-supplementary.pdf]

Supporting Information

Ultrastable and responsive foams based on 10-hydroxystearic acid soap for spores' decontamination

Carolina Dari[1], Fabrice Cousin[2], Clemence Le Coeur[2,3], Thomas Dubois[1], Thierry Benezech[1], Arnaud Saint-Jalmes[4*], and Anne-Laure Fameau[1*]

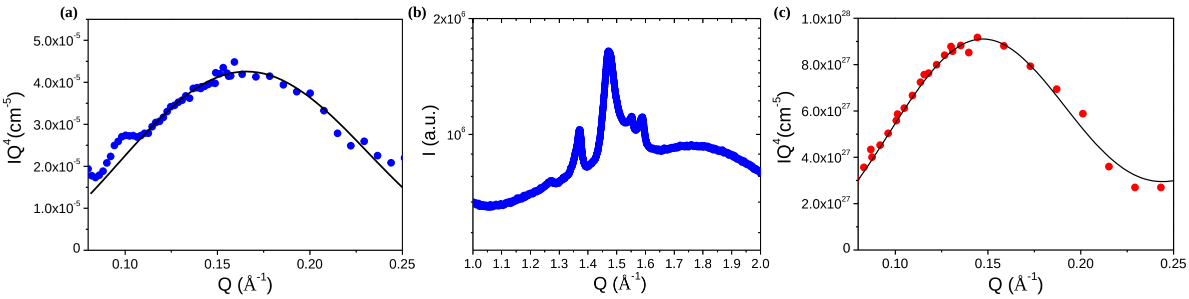

Figure S1. (a) SANS intensity profile of 10-HSA aqueous dispersion at 25 °C displayed in the Porod representation. The lines correspond to the best fit of the data using the model described in the literature [1]. (b) WAXS intensity spectrum of 10-HSA aqueous dispersion at 25 °C. (c) SANS intensity profile of 10-HSA aqueous dispersion at 75 °C displayed in the Porod representation. The lines correspond to the best fit of the data using the model described in the literature.

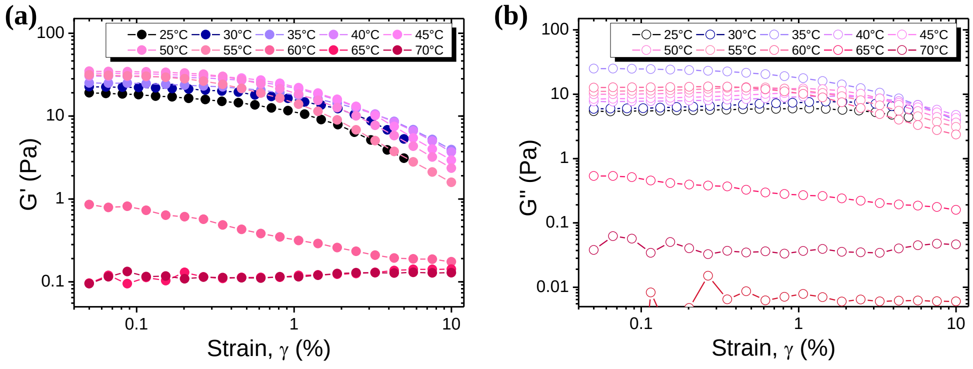

Figure S2. Oscillatory measurements of 10-HSA aqueous dispersion as a function of the strain amplitude, γ at constant $f = 1$ Hz at different temperatures: (a) elastic G' (●) and (b) viscous G'' moduli.

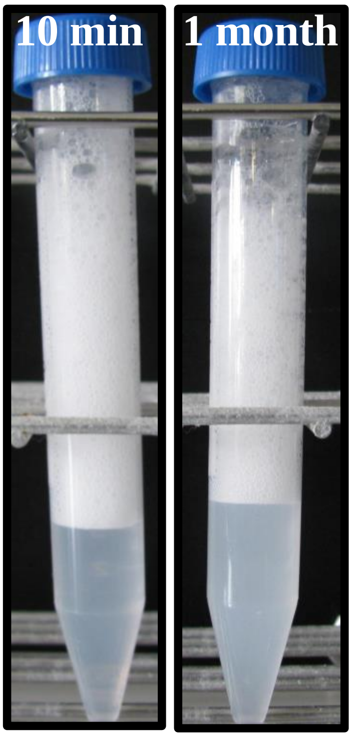

Figure S3. Pictures of 10-HSA foam produced by hand-shaking and kept at room temperature, just after foam formation (10 min) and after one month of conservation.

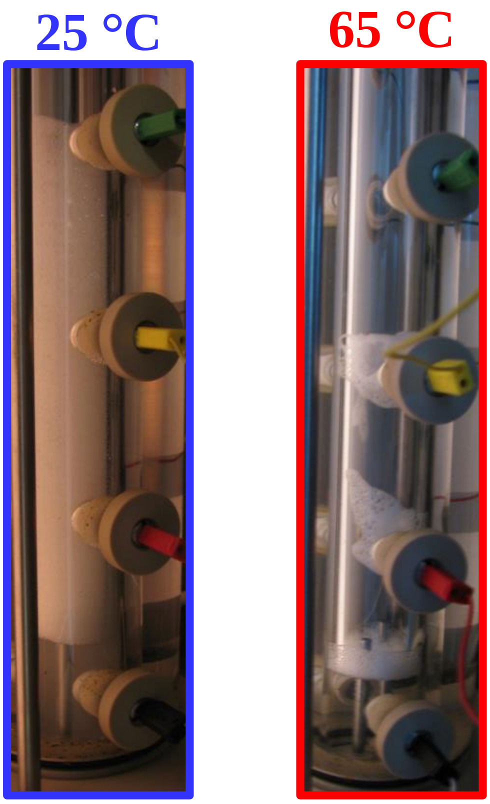

Figure S4. Pictures of the foam produced by bubbling gas using the Foamscan apparatus. Stable foam was obtained at 25°C, but by increasing the foam temperature at 65 °C, the foam was destroyed quickly in few minutes.

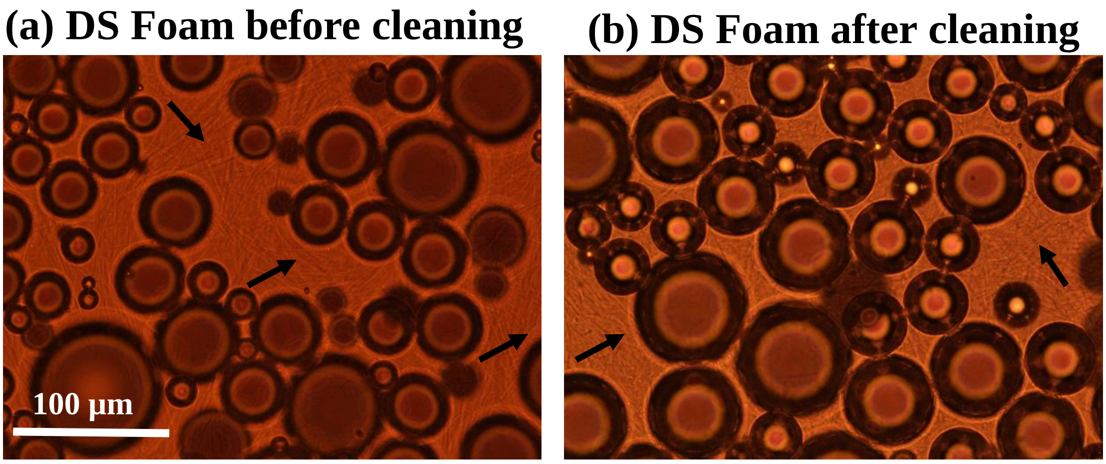

Figure S5. Pictures obtained by phase contrast microscopy of the double syringe (DS) foam: (a) before cleaning and (b) after cleaning. The arrows are pointing at some of the 10-HSA multilamellar tubes.

SANS data fitting:

The intensity scattered by objects in solution can be described as a product of structure factor S(Q), characteristic of the correlations between objects, by a form factor P(Q), that describes the shape of the objects. At large Q, the scattering curves scatters like Q^{-4} that is characteristic of a Porod behavior corresponding to the surface scattering of 3-D objects. The main equations used to fit the data of our system and consequently get further information on the objects in solution are those for lipids embedded in flat lamellar structures or micelles. For lamellar structure, the spectrum was fitted with a lamellar stack Caillé model used in SasView 5.0.4 (http://www.sasview.org/.), that is a random lamellar sheet with a Caillé structure factor. In this model of Caille S(Q) is used for the lamellar stacks. Through the form factor it is possible to describe some specific characteristics of the spectrum, namely the thickness of the membrane δ, with the function $P(Q) = \frac{2\Delta\rho^2}{q^2}(1 - cosq\delta)$.

We fixed the scattering length density of the solvent and the membranes (i.e. the fatty acids) and the total concentration, the others parameters were fitted. The line in the main text corresponds to the best fit of the data [1].

The form factor of a completely filled spherical object of radius R like micelles is given by :

Psphere(Q)=9[(sin(QR)-QRcos(QR))/(QR)^3]

For centrosymetrical objects like spheres, it is often considered that the structure factor S(Q) ~ 1 at large Q. Consequently, the radius of the micelle can be estimated from the form factor. The structure factor, S(q), which quantifies the intermicellar interactions/correlations, is included in our fitting process using the "Hayter-MSA-Structure" model displayed in the SASview software. This accounts for a repulsive screened Coulombic intermicellar interaction potential. The broad interaction peak observed in small angle neutron scattering spectra comes from the deprotonation of the COOH which introduces negative charges at the micellar/solvent interface. Six parameters are needed to compute the Hayter and Penfold structure factor: the

dielectric constant, the volume fraction, the effective radius of interacting objects, the temperature, the surface charge of the micelle, and the salt concentration. We fixed the dielectric constant, the volume fraction, the temperature, the scattering length density of the solvent and the sphere (in our case we took only the scattering length density of the fatty acid and not the MEA: Nb = -0.28*10-6 $Å^{-2}$), and the salt concentration. Thus, the micelle charge is the only fitting parameter for the calculation of S(q), and we found a value of -251. In the main text, the line corresponds to the best fit of the data according to the form and structure factor model. We observed the presence of negatively charged spherical micelles for all systems.

References:

[1] A.-L. Fameau, F. Cousin, A. Saint-Jalmes, Morphological Transition in Fatty Acid Self-Assemblies: A Process Driven by the Interplay between the Chain-Melting and Surface-Melting Process of the Hydrogen Bonds, Langmuir. 33 (2017). https://doi.org/10.1021/acs.langmuir.7b02651.
